# Complete dislodgement of a mechanical valve prosthesis: A rare but potentially life-threatening event: a case report

**DOI:** 10.1097/MD.0000000000038612

**Published:** 2024-06-28

**Authors:** Aijia Yu, Yunzhou Huang, Shutang Ren, Cuihua Wang, Jianhua Zhou, Taojun Ren

**Affiliations:** a Department of Ultrasound, TEDA International Cardiovascular Hospital, Tianjin, China.

**Keywords:** cardiovascular diseases, echocardiography, heart failure, heart valve prosthesis, transesophageal

## Abstract

**Rationale:**

Complete dislodgement of a mechanical valve is extremely uncommon as a long-term issue after getting a substitute mitral valve, and this report details an incident of complete detachment of a mechanical valve.

**Patient concerns:**

A 50-year-old woman, who underwent mitral mechanical valve replacement 2 decades earlier at another facility, was urgently admitted due to sudden cardiogenic shock.

**Diagnoses:**

Transthoracic echocardiograms revealed severe malfunction of the mitral valve prosthesis, characterized by significant mitral regurgitation and moderate pulmonary hypertension. Following the insertion of extracorporeal membrane oxygenation and an intra-aortic balloon pump, the hemodynamics stabilized. Coronary angiography displayed the prosthetic mitral valve ring and leaflet floating in the left atrium, as confirmed by preoperative real-time 3-dimensional transesophageal echocardiography. A complete separation of the prosthetic ring and leaflet from the suture ring was observed.

**Interventions:**

The patient promptly underwent bioprosthetic mitral valve replacement.

**Outcomes:**

The patient’s postoperative course was uneventful, leading to discharge in good condition.

**Lessons:**

A crucial aspect is comprehending the structure of the prosthetic valve itself. The use of transthoracic echocardiography and real-time 3-dimensional transesophageal echocardiography provides additional structural and functional details, enhancing support for potential life-saving interventions. Echocardiography plays a significant role in evaluating the morphology and function of prosthetic valves.

## 1. Introduction

Over 40 million individuals globally experience mitral or aortic valve disease, leading to a significant prevalence of heart valve replacements.^[1]^ Since its introduction in 1980, the bileaflet mechanical prosthetic heart valve has emerged as the predominant product due to its exceptional performance.^[2]^ Despite their increased durability compared to bioprostheses, mechanical prosthetic heart valves may encounter dysfunction attributed to 4 primary phenomena: thrombosis; fibrotic pannus ingrowth; degeneration; and endocarditis.^[3]^ Although complete valve dislodgement remains unreported, the primary causative factor is likely the deterioration and breakage of sutures. Acute dysfunction of prosthetic valves is a critical condition for any patient and may be linked to elevated mortality rates. In this report, we present a case involving a woman who experienced complete detachment of her mechanical valve over 2 decades after undergoing mitral valve replacement.

## 2. Case report

A 50-year-old woman was urgently admitted to the emergency department due to the abrupt onset of severe, progressive shortness of breath and cardiogenic shock. The patient had a medical history of mitral valve replacement with an unidentified prosthetic valve 2 decades earlier, performed at the age of 30 in another hospital, in response to a background of rheumatic heart disease. A transthoracic echocardiography (TTE) conducted 10 months prior had confirmed a normal left ventricular systolic function (ejection fraction: 55%) and proper functioning of the prosthetic mitral valve without pulmonary hypertension.

**Figure 1.**
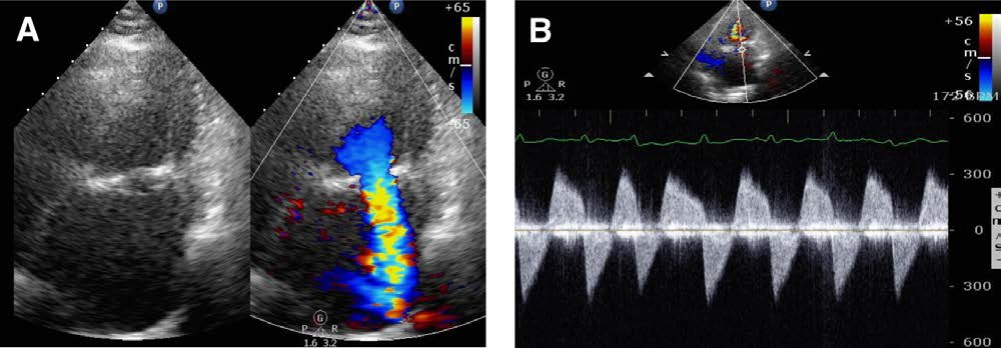
(A) In the TTE 4-chamber view, color Doppler flow indicated severe regurgitation due to prosthetic mitral valve dysfunction. (B) CW Doppler revealed a high-speed regurgitant flow into the left atrium. CW = continuous-wave, TTE = transthoracic echocardiography.

**Figure 2.**
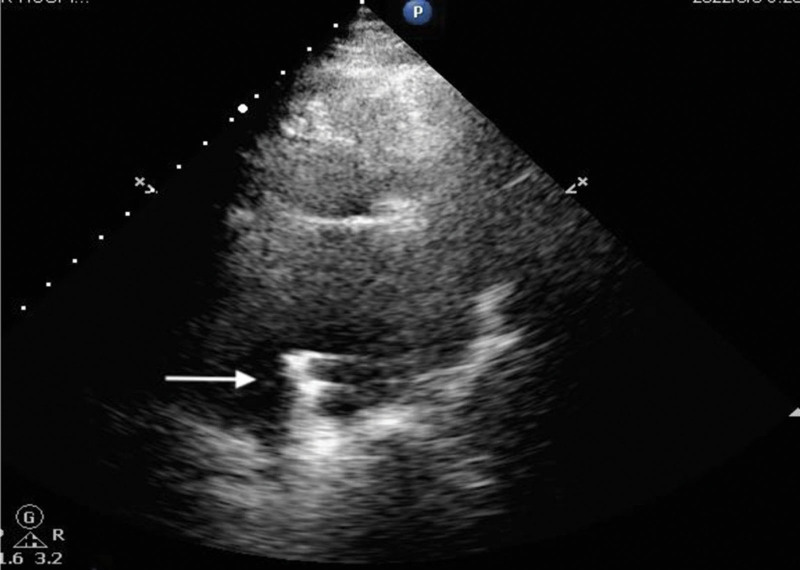
TTE displayed strong echogenic oscillations in the left atrium. TTE = transthoracic echocardiography.

**Figure 3.**
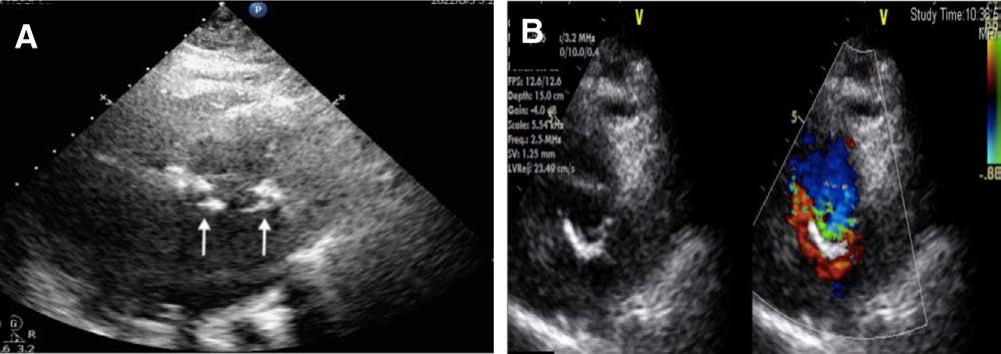
(A) Nonstandard apical 4-chamber views exhibited echoes consistent with a residual broken suture ring or endocardial appearance in the mitral position. (B) On the horizontal short-axis view of the mitral position, robust echoes resembling the prosthetic valve ring were still observed. However, A and B introduced some confusion and conflicting ultrasound information due to the limitations of TTE. TTE = transthoracic echocardiography.

**Figure 4.**
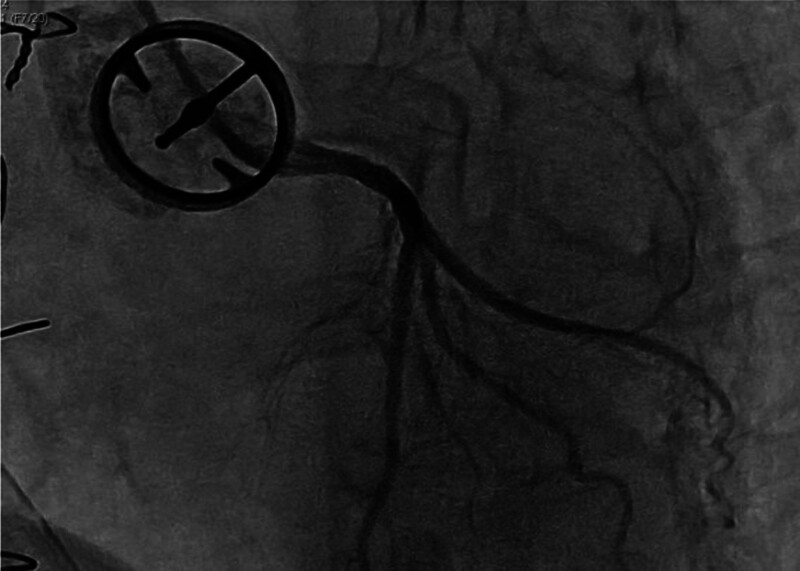
Coronary angiography illustrated the mitral prosthesis ring and leaflet floating in the left atrium.

**Figure 5.**
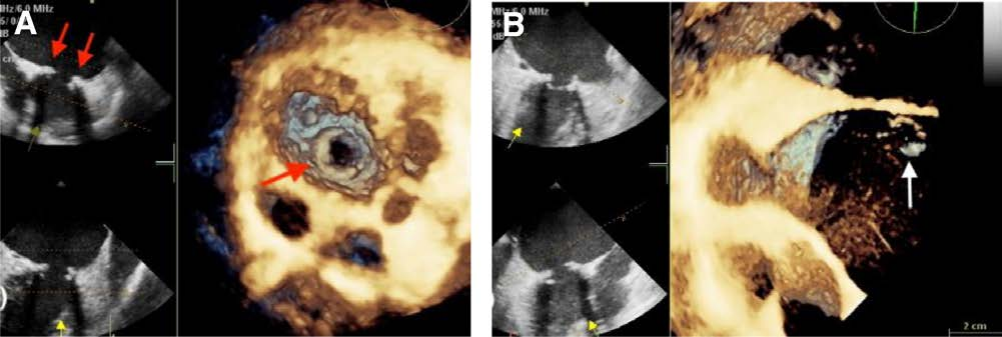
(A) Perioperative 3D-TEE images, looking from the left ventricle toward the mitral valve, depicted elevated tissue around the mitral position without forming a complete ring. (B) 3D-TEE revealed the complete prosthesis ring echo in the left atrium. 3D-TEE = three-dimensional real-time transesophageal echocardiography.

**Figure 6.**
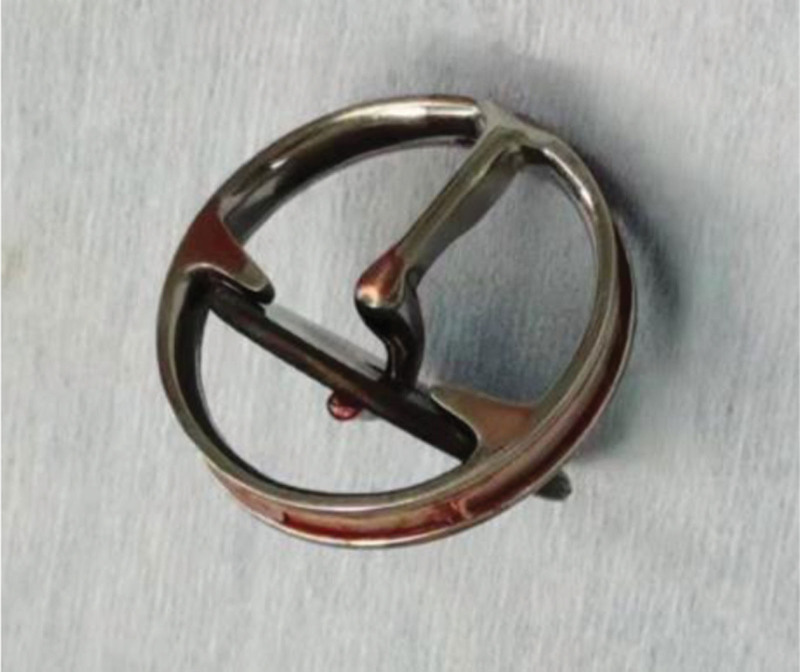
Surgeons identified a fully detached mitral prosthesis ring and leaflet in the left atrium.

Upon admission, the patient presented with acute and rapidly worsening dyspnea and lower limb edema, which had commenced 2 months earlier and intensified within the last hour. Clinical examination revealed tachycardia (175 beats/min), tachypnea (35 breaths/min), hypotension (82/65 mmHg), a body temperature of 35.6°C, and near-unconsciousness. Pulmonary auscultation disclosed diffuse rales in all lung fields. Electrocardiography indicated sinus tachycardia and atrial extrasystoles. An emergency bedside TTE was promptly performed to evaluate the cause of shock and acute decompensated heart failure. TTE affirmed the malfunction of the prosthetic mitral valve, displaying severe regurgitation (Fig. 1A) and moderate pulmonary hypertension (mean pulmonary artery pressure: 37 mm Hg). Continuous-wave Doppler illustrated a high-speed regurgitant flow into the left atrium (Fig. 1B), accompanied by abnormally robust echogenic oscillations within the left atrium (Fig. 2). The findings revealed complete mechanical failure of the mitral valve prosthesis, potentially resulting in the displacement of the prosthetic valve leaflet into the left atrium, causing significant left atrioventricular regurgitation and moderate pulmonary hypertension. However, due to the limitations of 2-dimensional ultrasound, there was some confusion and conflicting information. On nonstandard apical 4-chamber views, echoes consistent with a residual broken suture ring or endocardial appearance were observed in the mitral position (Fig. 3A). Conversely, on the horizontal short-axis view of the mitral position, strong echoes resembling the prosthetic valve ring were still visible (Fig. 3B). Notably, the characteristic ring-shaped echoes typical of a metal prosthesis ring were absent in the left atrium, suggesting possible dislodgement of the metal prosthesis leaflet without evidence of the prosthesis ring dislodgement. Following the placement of venoarterial extracorporeal membrane oxygenation which was initiated at 3 L/min flow and an intra-aortic balloon pump, cardiac function improved, and hemodynamics stabilized. Fraction of inspired oxygen was 80%. Coronary angiography revealed the mitral prosthesis ring and leaflet floating in the left atrium (Fig. 4). An emergency operation for bioprosthetic mitral valve replacement was performed. Intraoperative 3-dimensional real-time transesophageal echocardiography (3D-TEE) images indicated a bulge around the mitral position, possibly a broken suture ring or endothelial tissue, ruling out a complete metal prosthesis ring (Fig. 5A). The entire prosthesis ring echo was observed within the left atrium (Fig. 5B), further supporting the complete dislodgement of the metal prosthesis ring and leaflets. During the procedure, the surgeon discovered a broken prosthetic mitral valve fibrous suture ring, with no evidence of thrombosis or fibrotic pannus ingrowth. The mitral prosthesis ring and leaflet were found to have completely migrated into the left atrium (Fig. 6). The damaged suture ring was removed, and a successful bioprosthetic mitral valve replacement was carried out. Postoperative TEE demonstrated proper function of the mitral bioprosthesis. The patient regained consciousness on day 6 without the need for respiratory support. Postoperative TTE indicated well-functioning mitral valve with an ejection fraction of 60% and no pulmonary hypertension. The patient’s postoperative course was uneventful, leading to discharge after 16 days in good condition, with stable hemodynamic parameters, a normally functioning prosthetic valve on echocardiography, and effective anticoagulation.

## 3. Discussion

This case describes an uncommon complication following mechanical prosthetic heart valve surgery, specifically the dislodgement of the prosthetic ring and leaflet due to the deterioration of the fibrous component of the suture ring. Prompt and definitive diagnosis of complete mitral valve dysfunction was achieved through emergency bedside TTE. However, the 2-dimensional ultrasound’s technical limitations prevented the display of typical manifestations of the dislodged metal prosthetic ring’s echo in the left atrium, revealing only suspected leaflet dislodgement. Upon stabilizing hemodynamics with extracorporeal membrane oxygenation and intra-aortic balloon pump, coronary angiography revealed the drifting of the mitral prosthetic ring and leaflet in the left atrium. Preoperatively, 3D-TEE was employed to illustrate residual structures in the mitral position and disorganized strong echogenic oscillations within the left atrium. The surgical intervention involved removing the broken suture ring in the mitral position and replacing it with a bioprosthetic valve. Postsurgery, the mitral bioprosthetic valve functioned effectively, leading to the patient’s uncomplicated recovery and subsequent discharge. Mechanical valvular prostheses are preferred for younger recipients due to their durability. Prosthetic valve complications, both early and late, encompass PPM, geometric mismatch, dehiscence, primary failure, thrombosis, thromboembolism, pannus formation, pseudoaneurysm formation, endocarditis, and hemolysis.^[4]^ A study by Wang et al^[2]^ reported that Fuwai Hospital and Guangyuan First People’s Hospital conducted 24,710 valve replacements and 3264 first-time valve replacements over a decade. Among these cases, only 2 instances of unilateral leaflet escape were identified, constituting 0.06% of second-stage valve replacement cases. This shows that unilateral leaflet escape after mechanical valve replacement is a rare complication. Calik et al^[5]^ highlighted a case involving mitral leaflet escape and subsequent embolization to the left femoral artery in a young male patient, successfully addressed through multiple corrective surgeries. Kim et al^[6]^ reported a fractured escape of an Edward–Duromedics mitral valve occurring 27 years postsurgery. Lee et al^[7]^ documented a singular mitral leaflet escape in a 43-year-old man, who had undergone prosthetic aortic and mitral valve replacement 24 years earlier due to infective endocarditis. Abdallah et al^[8]^ reported a 54-year-old patient requiring repeated mitral replacement following a nontraumatic leaflet fracture of an Edwards Duromedics (Baxter) mechanical prosthesis, performed 33 years postimplantation. van Steenbergen et al^[9]^ described a case of spontaneous leaflet embolization 31 years after aortic valve replacement with an Edwards–Duromedics prosthesis.

Spontaneous leaflet fracture in mechanical heart valve prostheses is exceedingly rare, with documented cases primarily involving partial rather than complete dislodgement of the prosthetic valve ring and leaflets. However, our reported patient experienced a unique scenario where the complete displacement of the prosthetic ring and leaflet into the atrium resulted from the deterioration of the old broken fibrous component of the prosthetic suture ring. Notably, there are no prior reports documenting a similar occurrence.

Although a physical examination can alert clinicians to significant dysfunction in prosthetic valves, diagnostic methods are often necessary to assess their function. TTE with Doppler is the preferred initial noninvasive method for evaluating prosthetic valve function.^[4,10]^ When imaging prosthetic valves, it is crucial to examine the opening and closing motion of the moving parts, such as leaflets for bioprostheses and occluders for mechanical prostheses. Attention should also be given to identifying leaflet calcifications or abnormal echo density associated with the sewing ring, occluder, leaflets, stents, or cage. Additionally, a thorough inspection of the sewing ring is essential, focusing on potential separation from the native annulus and abnormal rocking motion during the cardiac cycle.^[4]^ In this case, understanding the design of the prosthetic valve is paramount. Currently, the most commonly implanted mechanical valves are bileaflet valves. These valves exhibit variations in the composition and purity of pyrolytic carbon, the shape and opening angle of the leaflets, the design of the pivots, the size and shape of the housing, and the design of the sewing ring.^[4]^ In the mitral position, mechanical prosthetic occluders exhibit no movement or shadowing, accompanied by abnormally strong echogenic oscillations in the left atrium during TTE. Determining the appearance of the sewing ring in the mitral position through two-dimensional (2D) ultrasound is challenging due to incomplete circular visibility in some views. However, strong echoes resembling the prosthetic valve ring are present in other views. To address this limitation, 3D-TEE proves valuable in identifying and clarifying information about mitral position echoes and strong echoes in the free state observed in 2D echocardiographic images. Particularly in the mitral position, TEE is more likely required than for native valves to evaluate prosthetic valvular structure and associated complications, notably regurgitation.^[4]^ For a comprehensive understanding of structures following detachment of the metal prosthesis ring and leaflets, 3D-TEE was employed preoperatively. This technique revealed the residual structure of the mitral position and the prosthetic ring displaced into the atrium. 3D-TEE images depict the sewing ring in the mitral position as an incomplete ring, and the imaging of a moving ring of strong echoes in the left atrium aligns with typical changes seen in 3D ultrasound images of metallic prosthetic rings. This suggests simultaneous detachment of the prosthesis ring and leaflet. 3D-TEE offers crucial additional information, and the amalgamation of 2D and 3D ultrasound images facilitates a more definitive diagnosis. In situations of unstable hemodynamic status, leveraging commonly used echocardiographic techniques like 2D, color Doppler, and spectral Doppler becomes crucial for prompt conclusions regarding prosthetic valve function. This includes assessing normal function, partial loss, or complete loss. Once hemodynamic stability is restored, preparing for surgical or interventional procedures allows for obtaining further structural and functional information using 3D-TEE. If needed, quantitative analysis techniques such as speckle tracking can be applied to garner additional structural and functional insights, offering comprehensive support for therapeutic measures. In summary, echocardiography plays a valuable role in evaluating both the morphology and function of prosthetic valves.

## Author contributions

**Formal analysis:** Aijia Yu.

**Writing**—**original draft:** Aijia Yu.

**Supervision:** Yunzhou Huang, Cuihua Wang.

**Writing**—**review & editing:** Yunzhou Huang, Shutang Ren.

**Resources:** Jianhua Zhou.

**Visualization:** Taojun Ren.
